# Long-term intra-individual variability of albuminuria in type 2 diabetes mellitus: implications for categorization of albumin excretion rate

**DOI:** 10.1186/s12882-017-0767-3

**Published:** 2017-12-06

**Authors:** Amanda Leong, Elif Ilhan Ekinci, Cattram Nguyen, Michele Milne, Mariam Hachem, Matthew Dobson, Richard J. MacIsaac, George Jerums

**Affiliations:** 1 Austin Health Endocrine Centre, Heidelberg Repatriation Hospital, PO BOX 5444, Melbourne, Victoria 3081 Australia; 2 Department of Medicine, University of Melbourne, Melbourne, Victoria Australia; 3 Menzies School of Health Research, Red 9, Casuarina Campus, University Drive North, Casuarina, Northern Territory 0811 Australia; 4 Murdoch Children’s Research Institute, Flemington Road, Melbourne, Victoria 3052 Australia; 5 Department of Endocrinology & Diabetes, St Vincent’s Hospital Melbourne, 41 Victoria Parade, Melbourne, Victoria 3065 Australia

**Keywords:** Diabetes, Diabetic nephropathy, Microalbuminuria, End-stage kidney disease, Albumin excretion rate, Diabetes mellitus type 2, Urinary excretion rate, Chronic kidney disease

## Abstract

**Background:**

Diabetic kidney disease (DKD) is the leading cause of end-stage renal disease in the Western world. Early and accurate identification of DKD offers the best chance of slowing the progression of kidney disease. An important method for evaluating risk of progressive DKD is abnormal albumin excretion rate (AER).

Due to the high variability in AER, most guidelines recommend the use of more than or equal to two out of three AER measurements within a 3- to 6-month period to categorise AER. There are recognised limitations of using AER as a marker of DKD because one quarter of patients with type 2 diabetes may develop kidney disease without an increase in albuminuria and spontaneous regression of albuminuria occurs frequently. Nevertheless, it is important to investigate the long-term intra-individual variability of AER in participants with type 2 diabetes.

**Methods:**

Consecutive AER measurements (median 19 per subject) were performed in 497 participants with type 2 diabetes from 1999 to 2012 (mean follow-up 7.9 ± 3 years). Baseline clinical characteristics were collected to determine associations with AER variability. Participants were categorised as having normo-, micro- or macroalbuminuria according to their initial three AER measurements. Participants were then categorised into four patterns of AER trajectories: persistent, intermittent, progressing and regressing. Coefficients of variation were used to measure intra-individual AER variability.

**Results:**

The median coefficient of variation of AER was 53.3%, 76.0% and 67.0% for subjects with normo-, micro- or macroalbuminuria at baseline. The coefficient of variation of AER was 37.7%, 66% and 94.8% for subjects with persistent, intermittent and progressing normoalbuminuria; 43%, 70.6%, 86.1% and 82.3% for subjects with persistent, intermittent, progressing and regressing microalbuminuria; and 55.2%, 67% and 82.4% for subjects with persistent, intermittent and regressing macroalbuminuria, respectively.

**Conclusion:**

High long-term variability of AER suggests that two out of three AER measurements may not always be adequate for the optimal categorisation and prediction of AER.

## Background

Diabetic kidney disease (DKD) is the leading cause of end-stage renal disease (ESRD) in the Western world. Current interventions for DKD do not arrest, but only delay the progression to ESRD [1]. The early and accurate identification of DKD, followed by early interventions may therefore offer the best chance of slowing the progression of kidney disease. One important method for evaluating the risk of progressive DKD involves identifying abnormal albumin excretion rate (AER), however the limitations of relying solely on AER as a marker of DKD are being increasingly recognised [2, 3].

The variability of 24 h urinary AER has been a topic for discussion due to its unpredictable nature. Rather than an abrupt transition from normal to abnormal values, albumin excretion often increases slowly over several years [4, 5]. The average increase in AER ranges from 10% to 30% per year until overt nephropathy develops, with some subjects showing slower rates of increase in AER after the development of microalbuminuria [6]. Regression from microalbuminuria to normoalbuminuria may also occur due to tight glycaemic control or the use of Renin-Angiotensin system inhibitors (RASi) [7]. Furthermore, the phenomenon of spontaneous regression from microalbuminuria to normoalbuminuria is now well recognised [5, 8–10]. Despite the above, the strong relationship of progression of albuminuria in type 2 diabetes mellitus (T2DM) to declining glomerular filtration rate (GFR), ESRD and cardiovascular (CV) disease emphasises the importance of accurately classifying AER patterns [11–13].

Several studies have demonstrated a wide range of intra-individual variability of albuminuria in diabetes, with the majority having a coefficient of variation in the range of 28% to 47% [5, 14]. Factors which affect the wide variation in AER include the type of urine sample analysed (e.g. 24 h, timed overnight, first morning, random), the concentration of urinary albumin, the time period over which the samples were collected (days, weeks, months), the clinical characteristics of participants, as well as the pre-analytical handling and storage of the urine samples [9, 14, 15]. The inherent variability of AER in people with diabetes also needs to be considered. Most previous studies investigating AER variability have been small and with a short follow up period in subjects with type 1 diabetes mellitus (T1DM) [16–18]. Only a few, small studies of the variability of albuminuria in T2DM have been previously reported [19, 20]. Furthermore, there is a deficiency of studies which have documented the variability of albumin excretion over a prolonged period, with the follow-up period in most studies being less than 1 year.

Due to the high variability in AER, most guidelines [14, 21] recommend the use of more than or equal to two out of three AER measurements within a 3- to 6-month period to categorise AER. Even with this recommendation, misclassification of AER categories can occur due to a potentially greater long-term variability of AER than that reported in shorter-term studies. The aim of this study was therefore to investigate the long-term intra-individual variability of albuminuria over several years. Furthermore, we sought to identify the relationship of the variability of AER with various clinical and biochemical parameters.

## Methods

### Study design

Consecutive AER measurements in 617 participants with T2DM were recorded from 1999 to 2012. These patients attended the diabetes clinics at Austin Health, Melbourne and provided 24 h urine samples prior to each clinic visit, at intervals of 3–12 months. Patients were asked to discard their first urine void of the day and collect urine for the next 24 h. AER was measured in each urine sample provided by the Department of Biochemistry at Austin Health. Over this time period, the Beckman method was used to measure urinary albumin. The laboratory coefficient of variation of this method is approximately 5%. We utilised a modified protocol based from Kania et al. [22] using a 10 ml aliquot of a 24 h urine collection, pH-adjusted with NaOH to a final concentration of 25 mM and stored at −20 degrees to prevent degradation of albumin.

Baseline clinical and biochemical characteristics were collected in 2000 (Table 1). These included sex, age, body mass index (BMI), disease duration, glycated haemoglobin (HbA1c), estimated glomerular filtration rate (eGFR), the use of RASi agents, smoking status, total cholesterol levels, high-density lipoprotein (HDL) cholesterol levels, and systolic blood pressure (SBP).

**Table 1:** Clinical characteristics according to baseline albuminuria group

	Normo (*n* = 289)	Micro (*n* = 157)	Macro (*n* = 51)
Gender (% male)	50.5	65.0	66.7
No. of AER measurements^a^	19 (8)	21 (8)	16 (8)
Duration of Follow-up (years)^a^	8.5 (3.0)	8.7 (3.1)	6.5(2.8)
ACEi/ARB use (%)	51.9	77.6	80.4
Smoking (%)	48.9	55.6	54.9
Age (years)^b^	66 (55, 73)	65 (58, 71)	64 (56, 69)
Diabetes duration (years)^b^	9.80 (5.36, 17.26)	11.40 (6.22, 17.29)	13.00(8.077, 21.41)
SBP (mmHg)^a^	139.4 (15.87)	142.39 (15.12)	143.58 (15.93)
BMI (kg/m^2^)^a^	29.10 (26.15, 33.33)	30.28 (27.11, 34.68)	32.11 (28.43, 34.21)
HbA1c (%)^a^	7.7 (1.4)	8.0 (1.4)	7.7 (1.8)
eGFR (ml/min/1.73m^2^)^a^	74.6 (19.25)	70.69 (19.25)	55.01 (22.25)
Total cholesterol (mmol/L)^a^	5.02 (0.99)	4.95 (0.92)	4.94 (1.13)
HDL-Cholesterol (mmol/L)^a^	1.17 (0.96, 1.4)	1.09 (0.88, 1.29)	1.01 (0.88, 1.19)

^a^mean ± SD

^b^median (IQR)

*ACEi/ARB* angiotensin converting enzyme inhibitor/ angiotensin receptor blocker

*SBP* systolic blood pressure

*HbA1c* glycated haemoglobin

*eGFR* estimated glomerular filtration rate

**Fig. 1:**
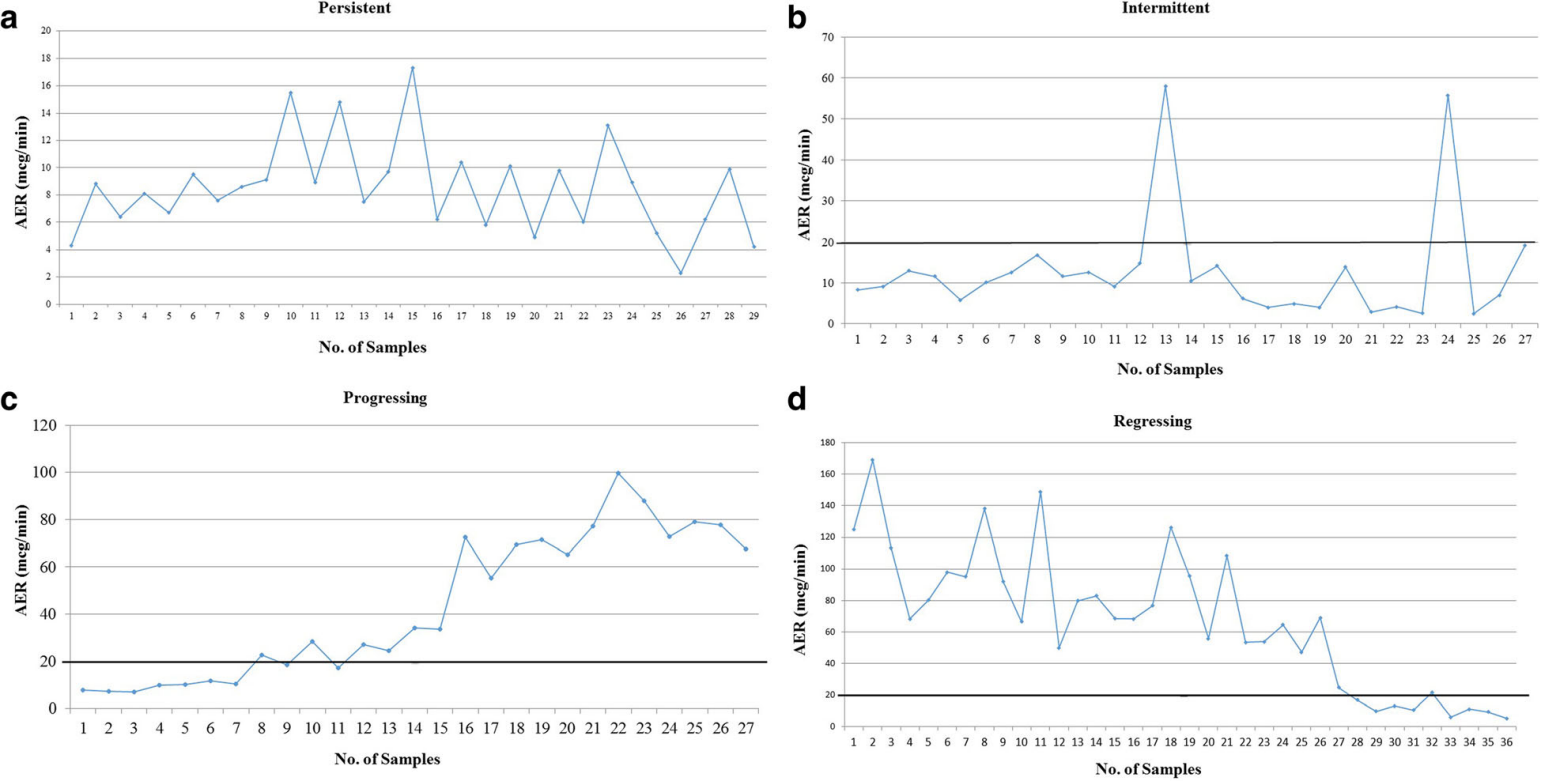
**a.** Persistent pattern (Normoalbuminuria): A representative plot of AER values for a patient where all AER values were <20 mcg/min throughout the study. **b.** Intermittent pattern (Microalbuminuria): A representative plot of AER values for a patient with occasional AER values >20 mcg/min (i.e., above the bold line), with AER values returning to baseline at study completion. **c.** Progressing pattern (normo- to microalbuminuria): A representative plot of AER values for a patient with AER <20 mcg/min at the start of the study progressing to >20 mcg/min at study completion. **d.** Regressing pattern (micro- to normoalbuminuria): A representative plot of AER values for a patient with AER >20 mcg/min at the start of the study, progressing to <20 mcg/min at study completion

A minimum of five AER measurements per subject was an inclusion criterion. Participants were first classified into baseline albuminuria categories: normo- (<20 mcg/min), micro- (20–200 mcg/min) and macroalbuminuria (>200 mcg/min) groups when at least two out of three of their first three samples fell within the respective ranges [3]. The first three samples were collected over an average period of 0.8 years. Participants were further independently subcategorized by two of the authors (AL and CN) into four AER pattern groups (Fig. 1):

**Persistent** (normo-, micro-, macro-): Participants with all AER values within the range of their respective baseline albuminuria categories.

**Intermittent** (micro-, macro-): Participants with one or more AER values above/below that of their respective baseline AER groups, and a return to their baseline albuminuria category at study completion (when at least two of the last three values were within the baseline category).

**Regressing**: Participants with a decrease in serial AER values, with two or more values in the range below their baseline categories at study completion.

**Progressing**: Participants with an increase in serial AER values, with two or more values above their baseline albuminuria categories at study completion.

These AER patterns were similar to those described in the study by Steinke et al. [23], which identified four patterns of AER trajectories (persistent, temporary persistent, intermittent and progression) in normo- and microalbuminuric patients with T1DM.

It should be noted that urinary albumin-creatinine ratio (ACR) was not measured in this study.

### Statistical analysis

The coefficient of variation is defined as the ratio of the standard deviation (SD) to the mean and was used as a measure of intra-individual AER variability. Median coefficients of variation of AER were used for each participant because the data were not normally distributed. The first three AER measurements were used to classify individuals into normo-, micro- and macroalbuminuria groups. Multivariate regression was used to examine the effects of baseline demographic variables on the intra-individual coefficient of variation of AER: baseline albuminuria group, HbA1c, age, gender, duration of diabetes, total cholesterol, HDL, systolic BP, BMI, RASi use at baseline and smoking. Multivariate regression was also performed to compare coefficients of variation among baseline albuminuria groups and AER pattern groups, with adjustment for clinical and biochemical characteristics. Wilcoxon rank-sum tests were performed to test the equality of coefficients of variation between those who have and those who have not had RASi treatment at baseline. All analyses were carried out using StataCorp. 2011. Stata Statistical Software: Release 12. College Station, TX: StataCorp LP.

## Results

### Patient characteristics

There were 617 potential participants with baseline and consecutive AER measurements. However 120 had insufficient follow-up AER data (i.e., less than five AER measurements) to permit categorization of temporal AER patterns. A total of 497 participants with sufficient AER data was therefore available for inclusion in the study. Participants were categorized into normo-, micro- or macroalbuminuria groups at baseline, and subsequently into one of the four longitudinal AER pattern groups.

Of the 497 participants in the study, 289 (58%) had normoalbuminuria, 157 (32%) had microalbuminuria and 51 (10%) had macroalbuminuria at baseline. The median number of urine samples for each of the 497 subjects was 19 (range 5–43) collected over 2–13 years. The number of samples was 19 ± 8, 21 ± 8 and 16 ± 8 (mean ± SD) for participants with baseline normo, micro or macroalbuminuria, respectively and the mean follow-up period for all participants was 7.9 ± 3 years. For those with baseline normo- micro- and macroalbuminuria, the follow up periods were 8.5 ± 3.0, 8.7 ± 3.1 and 6.5 ± 2.8 years, respectively. Baseline clinical characteristics according to albuminuria categories are shown in Table 1. As expected, a greater proportion of those who had micro- or macroalbuminuria at baseline were treated with RASi agents (normo 52%; micro 78%; macro 80%).

### Relationship between baseline AER variability and participant characteristics

Using multivariate regression, there was no evidence of a significant relationship between median coefficient of variation of baseline AER measurements for all participants and each of the following variables: HbA1c, age, gender, duration of diabetes, total cholesterol, HDL-cholesterol, SBP, BMI, and smoking.

The intra-individual variability of AER was compared between participants on (*n* = 312) and not on (*n* = 185) RASi agents at baseline, regardless of albuminuria group. The median coefficient of variation for AER was significantly higher in participants on RASi therapy at baseline compared to those not on RASi agents (66% vs. 55%, *p* = 0.003). After adjustment for baseline albuminuria categories, the coefficient of variation was 1.13 times greater in treated participants versus those not treated with RASi agents (*p* = 0.013).

At study completion, 98 out of 157 (62.4%) participants with microalbuminuria used RASi agents during the study. Eighty nine (90.8%) of the 98 participants were found to be on RASi treatment by the end of the study. For the remaining 9 participants, 6 were never treated with a RASi agent and 3 had ceased RASi treatment by the end of the study.

### Comparison of AER variability among normo-, micro- and macroalbuminuria groups

Coefficients of variation of each baseline albuminuria category are demonstrated in Table 2. After adjusting for baseline characteristics, the median coefficient of variation was 53% for participants with normoalbuminuria, 76% for those with microalbuminuria and 67% for those with macroalbuminuria. This coefficient of variation was significantly different among the three baseline albuminuria categories (*p* = 0.027). The median coefficient of variation was significantly lower in the normoalbuminuria group (67.7%) compared to the microalbuminuria group (81.6%) after adjustment for RASi use (*p* = 0.007). There was no evidence of a difference in coefficient of variation between the macroalbuminuria group (75.4%) and the normoalbuminuria group (*p* = 0.41).

**Table 2:** Albuminuria groups at baseline: median, mean and SD of the coefficient of variation of AER across the persistent, intermittent, progressing and regressing groups

Albuminuria group	Pattern	N	No. of samples mean (SD)	Median coefficient of variation	Mean coefficient of variation	SD	*p*-value*	*p*-value†
Normoalbuminuria	Total	289	19 (7)	53.3	67.7	0.450		
	Persistent	116	18 (7)	37.7	38.2	0.121		
	Intermittent	111	20 (8)	66.0	78.8	0.474	<0.001	
	Progressing	62	20 (8)	94.8	100.3	0.459	<0.001	
Microalbuminuria	Total	157	19 (6)	76.0	81.6	0.38	0.007	
	Persistent	5	15 (2)	43.0	41.3	0.103		
	Intermittent	84	21 (8)	70.6	74.8	0.279	0.064	
	Progressing	28	22 (7)	86.1	98.8	0.531	0.002	0.003
	Regressing	40	22 (8)	82.3	89.1	0.399	0.008	0.033
Macroalbuminuria	Total	51	15 (8)	67.0	75.4	0.439	0.41‡	
	Persistent	18	13 (4)	55.2	61.8	0.264		
	Intermittent	15	17 (10)	67.0	69.2	0.218	0.071	
	Remission	18	17 (9)	82.4	94.1	0.631	0.071	

‡P-values result from multivariate regression between each albuminuria group and the normoalbuminuria group

*P-values result from multivariate regression between each pattern and the persistent pattern of each respective albuminuria group

†P-values result from multivariate regression between each pattern and the intermittent pattern of each respective albuminuria group

### Comparison of AER variability for temporal AER patterns according to baseline AER

#### Normoalbuminuria at baseline

Coefficients of variation of the persistent, intermittent and progressing patterns of AER are demonstrated in Table 2. There was a difference in coefficients of variation across the three patterns after adjustment for baseline characteristics (*p* < 0.001). The median coefficient of variation was significantly lower in the persistent pattern (38%) than the intermittent pattern (79%; *p* < 0.001), and also lower when compared to the progressing pattern group (95%; *p* < 0.001) by definition as AER was changing.

#### Microalbuminuria at baseline

Coefficient of variations of the persistent, intermittent, progressing and regressing patterns of AER for participants with baseline microalbuminuria are shown in Table 2. There was an overall difference in coefficients of variation across the four AER patterns after adjusting for baseline characteristics (*p* = 0.001). The median coefficient of variation was similar in the persistent pattern (43%) compared to the intermittent pattern (71%; *p* = 0.064), but lower than the progressing pattern (86%; *p* = 0.002) and the regressing pattern (82%; *p* = 0.008) groups. There was also evidence of a difference in coefficients of variation between the intermittent and progressing pattern groups (*p* = 0.003), as well as a difference between the intermittent and regressing pattern groups (*p* = 0.033). In the present study, 28% of participants showed remission from micro- to normoalbuminuria independently of RASi use.

#### Macroalbuminuria at baseline

Coefficients of variation of the persistent, intermittent and regressing patterns are shown in Table 2. There was no evidence of a significant difference in coefficients of variation among the three AER patterns (*p* = 0.071).

### Comparison of variability of AER according to temporal patterns of AER with or without RASi treatment

There was no evidence of a difference in the coefficient of variations of AER amongst the patients treated with or without RASi with any of the temporal AER patterns.

### Theoretical effect of increasing AER measurements

**Fig. 2:**
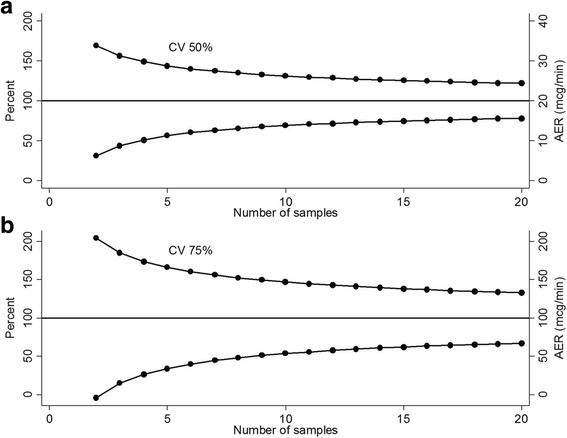
Panel **a**: (*top*) Theoretical plot for intra-individual coefficient of variation in the normoalbuminuria group with 95% confidence intervals for 2–20 samples: coefficient of variation = 50%, mean AER = 20 mcg/min. Panel **b**: (*bottom*) Theoretical plot for intra-individual coefficient of variation in the microalbuminuria group with 95% confidence intervals for 2–20 samples: coefficient of variation = 75%, mean AER = 100 mcg/min

The theoretical effect of increasing the number of AER measurements per participant on 95% confidence limits for intra-individual coefficient of variations of AER arbitrarily set at 50% (normoalbuminuria) and 75% (microalbuminuria) respectively is shown in Fig. 2. If a patient with normoalbuminuria at baseline provides three urine samples with a median AER of 20 mcg/min and a coefficient of variation of 50%, the 95% confidence interval would be approximately 50–150%. With seven or more samples, the confidence interval plateaus at approximately 60–130%. Similarly, if a participant with microalbuminuria at baseline provides three urine samples with a median of 100 mcg/min and a coefficient of variation of 75%, the 95% confidence interval would be approximately 20–180%. With ten or more samples, the confidence interval plateaus at approximately 50–150%. It appears that a larger number of AER measurements are needed for participants with microalbuminuria than normoalbuminuria in order to achieve similar confidence intervals.

## Discussion

The major finding of this study is that the long-term intra-individual coefficient of variation of AER is high, implying that more than three AER measurements may be necessary to accurately categorise albuminuria. In this study, we categorized albuminuria into normo-, micro- or macroalbuminuria groups according to participants’ initial three AER measurements and then subsequently into groups according to four patterns of AER trajectories: persistent, intermittent, progressing and regressing. The coefficient of variation of AER was 37.7%, 66% and 94.8% for subjects with normoalbuminuria who had persistent, intermittent and progressing AER pattern. It is recognised that by definition, patients with persistent AER measurements within the normo-, micro- and macro-albuminuria groups will have a lower co-efficient of variation than patients with intermittent, progressing or regressing patterns of AER. The coefficient of variation of AER for micro- and macroalbuminuria patients, regardless of the subsequent AER pattern fell within the above boundaries. In addition, out of all the baseline clinical and biochemical characteristics analysed, only the use of RASi agents significantly influenced the variability of AER. However, this effect was only evident for baseline AER variability and the data were insufficient to estimate the influence of RASi on the long term temporal patterns of AER used in this study. Kropelin et al. [24] evaluated data from three randomized intervention trials (BENEDICT, DIRECT, ALTITUDE and the Irbestartan in Patients with Type 2 Diabetes and Microalbuminuria (IRMA-2)) study and demonstrated that increasing the number of urine collections per study visit and the number of visits does not change the average drug effect estimate. It was therefore suggested that using a single urine collection per study visit was sufficient to define transition of albuminuria as an end-point in clinical trials [24].

The median intra-individual coefficients of variation of AER reported here are higher than the intra-individual variability seen in previous studies in T2DM (31–43%) [4, 20, 25]. These previous studies were typically conducted under stricter conditions (i.e., as part of a clinical investigation and not an outpatient setting). Although, one study conducted under routine clinic conditions, involving 1391 participants with T1DM and T2DM, in which an average of 2 to 3 samples per participant per visit (range 2 to 18) were collected over 2 years, reporting a coefficient of variation of 58%–82% [26]. The differences in coefficients of variation between our study and previous reports are likely to be multi-factorial. Many of the studies mentioned were conducted over shorter time intervals, on smaller numbers of patients, with smaller numbers of urine samples collected per participant and in specific study settings. By contrast, the current study investigated the intra-individual variability of AERs for patients with T2DM undergoing routine clinical assessment and a mean follow up of 7.9 years [4, 25].

There have only been a few previous reports of AER variability for patients with T2DM according to baseline AER categories of normo-, micro- and macroalbuminuria. In one study of 87 participants where only 3 overnight samples were collected per participant, the overall variability of AER was 25.7%, compared with 36.1%, 24.8% and 22.3%, for normo-, micro- and macroalbuminuric participants, respectively [27]. In contrast, the present study showed that the median coefficient of variation was significantly lower in those with normoalbuminuria (53.3%) compared to those with microalbuminuria (76%) after adjustment for the use of multiple characteristics including RASi agents (*p* = 0.007) at baseline. This study also showed a median coefficient of variation of 67% for those with macroalbuminuria. The higher intra-individual coefficient of variation in the albuminuria groups in the current study could be attributed to the longer duration of this study and the larger number of samples collected. Other factors that could have contributed include the progression of disease, effect of treatment on the disease or a fall in the number of participants as the years progressed.

The Renal Insufficiency and Cardiovascular Events (RIACE) study has also reported on the variability of albuminuria in T2DM [20]. The investigators determined AER from a subset of participants - 833 subjects had AER and 3229 participants had ACR measured at different laboratories. These investigators determined that the concordance rate between a single urinary albumin excretion (UAE) and geometric mean of multiple measurements depended upon the degree of albuminuria- 94.6% for normoalbuminuria, 83.5% for microalbuminuria, and 91.1% for macroalbuminuria. It is difficult to directly compare our results with the RIACE study, as the RIACE study used both ACR and AER, whereas the current study used AER. In the RIACE study, only 20.5% of participants had an AER measurement and 79.5% had an ACR measurement. Another limitation of the RIACE study is that participant characteristics were not equally distributed among albuminuria classes. In the RIACE study, the distribution of participants according to AER categories was 71.9% with normoalbuminuria, 23.2% with microalbuminuria and 4.9% with macroalbuminuria. In our study, we had 58% with normoalbuminuria, 32% with microalbuminuria and 10% with macroalbuminuria. Furthermore, compared to the RIACE study, we used AER measurements in all our participants. It is possible that using ACR can alter the number of samples required to categorise albuminuria, however in the current study our main purpose was to demonstrate AER variability.

An important aim of the present study was to observe temporal AER patterns and to determine baseline predictors of those patterns. Interestingly, in the current study, we found that only 5 out of 157 participants with microalbuminuria at baseline were persistently microalbuminuric throughout the average of 7.9 ± 3.1 years follow up period. The current study highlights the highly variable nature of microalbuminuria in patients with T2D. Historically, it has been assumed that the development of microalbuminuria signalled the inevitable progression to macroalbuminuria [14]. However, there is an increasingly recognised view that the development of microalbuminuria can no longer be viewed as a committed and irreversible stage of DKD, with spontaneous remission being frequently reported [2, 11]. Approximately 60% of patients with T1DM have displayed spontaneous remission of microalbuminuria independent of the use of RASi agents over 5–10 years of follow up [14]. However, spontaneous remission of microalbuminuria to normoalbuminuria in a cohort study of T1D patients followed up for over 30 years was not associated with a reduction in CV or renal risk compared to sustained microalbuminuria despite adjustment for RASi inhibitors [13]. However, the lack of association between CV and renal risk in the remission of microalbuminuria to normoalbuminuria may have been missed [28]. Changes in albuminuria may have been too small to detect any clinical significance despite long-term follow-up. In the present study of participants with T2DM, 28% showed remission from micro- to normoalbuminuria independent of RASi agent use. Other studies of T1DM and T2DM have reported rates of spontaneous remission from microalbuminuria to normoalbuminuria that have ranged from 39%–64% [9, 16, 17, 23].

We found that the baseline characteristics of sex, age, BMI, disease duration, HbA1c, smoking status, total cholesterol levels, HDL-cholesterol, SBP, and even use of RASi agents had no significant effect on the variability of AER. One limitation of this study was the inability to compare changes over time in medications, HbA1c, eGFR, systolic blood pressure and cholesterol/HDL levels with the changes over time in AER. The definitive answer as to whether a relationship does or does not exist between clinical and biochemical parameters and variability in AER therefore requires a study to determine if there is a relationship between temporal changes in both clinical and biochemical parameters over time and variability in AER. Unfortunately, we were not able to account for factors such as blood pressure, glycaemic control, dietary salt consumption, physical activity and inflammation as information pertaining to the above variables was not collected in a longitudinal fashion for this study. We recognise this as a limitation of the current study. Despite this, several studies have shown that there is no association between the variability of AER and sex [18, 25], age [18], BMI [18], total cholesterol [18, 19, 29] and SBP [18].

In this study, we also examined the theoretical effect of increasing the number of AER measurements per participant on 95 confidence limits for intra-individual coefficient of variation. As seen in Fig. 2, seven to ten measurements of AER can mark the beginning of the plateau of the 95% confidence intervals for the normoalbuminuric and microalbuminuric groups, respectively. It can therefore be inferred from the current study that the commonly accepted definition of two out of the first three samples is insufficient to categorize albuminuria at baseline (3). The RIACE study also suggests that although two AER samples can provide a robust classification of albuminuria status (sensitivity of 90.6 and specificity of 94.6), disease progression and the efficacy of reno-protective treatment such as the use of RASi agents cannot be accurately monitored unless albuminuria is measured at frequent intervals over a prolonged period of time [20]. Similarly, 388 T2DM patients using RASi inhibitors, losartan or irbestartan in the RENAAL and IDNT trials, showed a 30% reduction in albuminuria 3 months after commencement of RASi inhibition and a further decrease of 44.8% in 174 patients after 12 months [30]. The variability of albuminuria within patients suggests that incorporating multiple measurements improves risk algorithms and assessment of treatment effects over time [30].

In this study, 120 out of 617 (19%) participants were excluded from the study due to the lack of sufficient AER samples collected (minimum of five samples were required to be included in the study). This significant portion of participants excluded is a potential selection bias as patients with less samples collected were potentially those who are poorly compliant with follow up requirements. A limitation to the current study and to studies investigating albuminuria, is that there is no reference laboratory method of measuring urine or serum albumin levels [31]. Albuminuria is calibrated to a serum albumin reference material and is diluted to the concentrations measured in urine [31]. There is also a lack of development of a standard procedure for dilution and diluent in measuring albuminuria [31]. Furthermore, in the current study, we focused on albumin excretion rate as it has traditionally been accepted as the reference method for determining albuminuria rather than the albumin to creatinine ratio and did not specifically study albumin to creatinine ratio in the current study. However, it is appreciated that the albumin to creatinine ratio is usually now the preferred method for assessing albuminuria.

It is appreciated that it is often impractical to obtain multiple AER measurements in the clinical setting before deciding on treatment. Furthermore, it is important to appreciate that the relationship between AER and renal/vascular outcomes is continuous [5]. Attempts to classify AER into categories are performed to provide a simple framework for researchers and clinicians to interpret the results of interventions that alter albuminuria and to stratify the risk that individual patients have for the development and progression of renal and vascular disease. Clinicians should be aware of the wide variability of urinary albumin excretion and find a balance between theoretical suggestions for the number of AER measurements in the context of other risk factors. For instance, increases in AER in participants with hypertension are an indication for intervention whereas short-term increases in AER in normotensive participants are not necessarily an indication for intervention. During the time that the current study was conducted, many centres were using AER routinely as the way of determining albuminuria. Over the years, ACR has increasingly been used to determine albuminuria. The current study addressed the variability in AER therefore we are not able to comment as to whether the study will change the way we manage our patients as very few centres are now using AER to measure albuminuria.

## Conclusions

The current study highlights an important finding, that there is a high degree of variability of AER in people with diabetes and that this high long-term variability of AER suggests that two out of three AER measurements may not always be adequate for the optimal categorisation and prediction of AER.

## Abbreviations

ACR: Albumin-creatinine ratio
AER: Albumin excretion rate
BMI: Body mass index
CKD: Chronic kidney disease
CV: Cardiovascular
DKD: Diabetic kidney disease
eGFR: Estimated glomerular filtration rate
ESRD: End stage renal disease
GFR: Glomerular filtration rate
HbA1c: Glycated haemoglobin
HDL: High-density lipoprotein
RASi: Renin-angiotensin system inhibitor
RIACE: Renal insufficiency and cardiovascular events study
SBP: Systolic blood pressure
SD: Standard deviation
T1DM: Type 1 diabetes mellitus
T2DM: Type 2 diabetes mellitus
